# Repeal of the Pennsylvania motorcycle helmet law: reflections on the ethical and political dynamics of public health reform

**DOI:** 10.1186/1471-2458-10-202

**Published:** 2010-04-21

**Authors:** Robert A Cherry

**Affiliations:** 1 Surgery and Public Health Sciences, Penn State Shock Trauma Center, MC H075, Penn State Milton S Hershey Medical Center, 500 University Drive, Hershey, Pennsylvania 17033, USA

## Abstract

**Background:**

In June of 2003 the Commonwealth of Pennsylvania passed S. 259 which repealed the state's 35-year old motorcycle helmet safety law. Motorcycle helmets are now only required for riders who are under the age of 21 and for those who are 21 years or older who have had a motorcycle operator's license for less than two years, or who have not completed an approved motorcycle safety course.

**Discussion:**

Prior to the repeal, and in the years that have followed, there has been intense debate and controversy regarding Pennsylvania's decision to repeal the law that required universal and mandatory use of motorcycle helmets for all riders. Proponents of the helmet repeal have argued in favor of individual rights and freedom, whereas advocates for mandatory helmet laws have voiced concerns over public health and safety based on available data.

**Summary:**

This commentary will discuss the policy-making process that led to Pennsylvania's repeal of the motorcycle helmet safety law from an ethical, political, and economic perspective.

## Background

Just prior to the repeal of the Pennsylvania motorcycle helmet law in 2003, the National Highway Traffic Safety Administration (NHTSA) released a number of interesting findings that are worth mentioning: 1) there were 3,244 motorcycle deaths and 65,000 injuries in 2002 on US highways; 2) motorcyclists are 27 times more likely to die in motorcycle crash (MCC) per mile traveled than an occupant in an automobile; 3) head injury is the leading cause of death for MCC; 4) motorcycle helmets reduce the likelihood of death in a MCC by 37%; 5) the Crash Outcome Data Evaluation System (CODES) study demonstrated that motorcycle helmets are 67% effective in the prevention of traumatic brain injury and; 6) motorcycle helmet use saved $1.3 billion in 2002 and an additional $853 million could have been saved if helmets were worn in all MCC.

In the face of such compelling data, why did Pennsylvania repeal its motorcycle helmet safety law? In 2002, there were 134 motorcycle-related crash fatalities. After the repeal of the law, this number increased to 205 in 2005. Is this an example of public policy gone awry?

## Discussion

Anti-helmet advocates argued for the repeal of the Pennsylvania motorcycle helmet law for several reasons: 1) freedom of choice and individual rights, 2) the pleasure of riding without a helmet, and 3) helmet use increases the odds of spinal cord damage. In response, the American Insurance Association and Western Pennsylvania's Hospital Council opposed the repeal and warned that "employers, consumers and health care providers will pay a price through the loss of productivity due to more people being involved in more severe crashes and the attendant increase in medical costs" [1]. Medical experts stated that there is no evidence that helmets increase your chances of spinal cord injury during a MCC. American Bikers Active Towards Education (ABATE) has been fighting for optional helmet laws and has said, "From our perspective, we still think it should be a matter of adult choice" [2].

Public health officials have long favored motorcycle helmet safety laws because they are designed to protect lives and reduce the impact of economic losses due to injury or death. From an ethical frame, public health officials and health care professionals who share this opinion tend to be objective utilitarians. These individuals are advocates for the well-being of individuals in society but are not necessarily confident that people can make reliable and valid choices.

Objective utilitarians argue that decisions regarding individual well being should be objectively defined by a group of experts. In this case, motorcyclists should wear helmets when operating their bikes because, according to published scientific studies, this practice has been shown to saves lives. The argument is further refined and supported through the use of tools that evaluate the cost-effectiveness of a policy decision. Mandatory motorcycle helmet use is a cost-effective measure, relative to the economic losses resulting from health care costs and the temporary or permanent loss of employability, if a motorcyclist is injured or killed during a crash. Helmets are still a relatively inexpensive public health preventative measure. Of note, proponents on both sides are not particularly concerned that there is a misallocation of the economy's resources with respect to helmet cost and distribution.

Some might argue that the classification of motorcycle health proponents as objective utilitarians is an oversimplification and might misrepresent their potentially diverse perspectives on the issue. The reasons for supporting motorcycle helmet laws may be varied and advocates may not necessarily share a common vision for achieving social utility. John and Bayer, for instance, would categorize proponents for helmet laws as paternalists rather than utilitarians [3]. Paternalists would support state regulated behavior requiring motorcycle helmets if the social and economic burden resulting from helmetless motorcycle fatalities and injuries is not in the best interest of society.

So what is at the heart of the policy debate? Anti-helmet advocates fundamentally define the problem as an issue of freedom of choice and, therefore, argue that such freedoms should be afforded protections under the law. Libertarians, as they are called, generally oppose governmental actions that restrict individual freedom of choice. Anti-helmet advocates believe that the restrictions placed on them by mandatory helmet use infringes on their individual rights and should therefore be repealed. This perspective is not very different from many of those who smoke tobacco. Some smokers argue that their individual rights are violated when government or businesses enforce tobacco free zones in certain areas, such as restaurants, bars, or the property that a hospital occupies. Objective utilitarians and paternalists argue that there is evidence that second hand smoke is a dangerous environmental concern. Those in close proximity to second hand smoke should be protected from potential harm. In the case of motorcycle helmet use, however, there are no environmental issues and their choice to not wear a helmet does not pose a physical threat or danger to anyone else. On the other hand, the socioeconomic costs related to medical expenses, insurance costs, lost earnings and wages, unemployment compensation, and disability might constitute harm to society as a whole, extending beyond the individual who chose not to wear a helmet during a motorcycle crash.

As a result of this perceived problem, a 'diagnostic' approach was performed by anti-helmet advocates to evaluate how this system could be reformed or 'cured'. They chose to focus on two control knobs involved in health-sector reform: regulation and behavior. Libertarians generally oppose government regulation because such statutes are believed to be a form of coercion and used to alter behavior and freedom of choice. In this situation, public health regulations were used to influence behavior through laws that require motorcycle helmet use.

Once the problem was diagnosed, anti-helmet advocates then had to turn towards policy development. They did not have to look too far for innovative thinking. In some cases, policy reformers must seek out new ideas internationally, or outside of the public health sphere, or even examine theoretical sources for their paradigm shift. For anti-helmet advocates, there were already a number of states that were setting precedents by easing restrictions on motorcycle helmet use. Arkansas, Texas, Kentucky, Louisiana, and Florida weakened laws governing universal helmet use and limited coverage to those less than 21 years old. (NHTSA, April 2004). Objective utilitarians argued that MCC fatalities increased 21% and 31% in Arkansas and Texas respectively in the first full year following repeal of these laws. In Florida, there was an 81% increase in motorcycle fatalities in the three years after the state's helmet law was repealed in 2003. At the time, the media made comparisons to a 2003 federal review demonstrating that fatalities increased over 50 percent in Kentucky and 100 percent in Louisiana after helmet laws were repealed in those states [4].

In addition to concerns regarding the cost-effectiveness of motorcycle helmet use, objective utilitarians and paternalists also argued that the quality adjusted life years per dollar spent on helmets was a worthwhile investment.

Simply borrowing policies from other states was not enough. Reformers also had to look ahead and draft a policy with the appropriate language and content that would allow for a favorable political decision. Anti-helmet advocates were very much aware of the MCC injury and fatality data that was disseminated from those five states that repealed their motorcycle helmet laws. They argued that many of these crashes were due to the inexperience of the rider and sought to weaken the argument of objective utilitarians by introducing language that would require helmets for individuals over the age of 21 who did not have a valid license for at least two years, or did not take an approved motorcycle safety course. One could almost say that the anti-helmet advocates took a page from the National Rifle Association's slogan: "Guns don't' kill people. People kill people". In this case, one could argue, 'Helmets don't save people. People save people'. The group was very effective at performing a stakeholder analysis of the vested interest groups, individuals, parties, and organizers. This allowed them to draw a political roadmap to success and draft a Best Alternative to Negotiated Agreement. In other words, if negotiations are not successful, and an agreement cannot be obtained, then a party will take the best course of action or best alternative to a negotiated agreement.

Another important aspect in re-designing the policy was the process by which anti-helmet advocates took to get there. This allowed for the mobilization of key allies needed to support the reform process. For example, ABATE is a strong political force, both regionally and nationally. ABATE "is an organization of motorcyclists dedicated to the protection of the individual rights of motorcyclists through political change, public education, and charitable works," according to its website [5]. There are 7,000 members in the state of Pennsylvania alone. The organization has chapters across the United States and is considered a state motorcycle rights organization (SMRO). ABATE conducted an annual Lobby Day at the state capitol in Pennsylvania and an annual Rights Rally Day. ABATE Chapters work with national motorcycle organizations such as the American Motorcycle Association and the Motorcycle Riders Foundation. In addition, the Pennsylvania Chapter of ABATE employs a full time lobbyist. Motorcyclists are a powerful political action group with a number of high profile elected officials invited to speak at their Annual Rights Day Rally.

The ethical frame was therefore muted by four political factors at play in health-sector reform: 1) Players - those individuals and groups involved in the policy reform process, 2) Power - the relative power that each player has in the political arena, 3) Position - the position expressed by each player and the intensity and proportion of resources devoted to that position, and 4) Perception - the public perception of the problem and proposed solution to that problem [6]. The anti-helmet advocates had a clear political advantage if you look at the players involved, the power of each player, and intensity and resources devoted to the reform process. Public health officials and health care professionals have historically been disadvantaged as an organized political force relative to groups such as this, especially as it relates to players, power, and position in the political arena.

Jones and Bayer also argued that there was a long-standing inability on the part of public health officials to successfully defend and justify paternalistic protective legislation, which "is aimed at protecting the people from self-imposed injuries and avoidable harms" [3]. The challenge for public health, according to the authors, is to overcome the strong sense of individual liberty and choice that is part of American political culture.

The political feasibility of policy reform, and the ability to win a political decision that favors helmet use, therefore largely depended on the ability of the public health community to change public perception. From the perspective of an elected official, there is probably little risk in siding with the position outlined by the motorcycle lobbyists. The overwhelming majority of motorists do not ride a motorcycle and, relative to other political issues, motorcycle helmet use is probably not a particularly high-ranking pocketbook or sociopolitical issue. It is unlikely that an automobile driver will alter his/her vote based on the position taken by their local representative with respect to helmet use. Therefore, altering public perception that motorcycle helmet use is an important issue among automobile drivers is probably a challenging task. On the other side of the equation, motorcyclists represent a sizable portion of the voters. In 2003, for example, there were 755,068 licensed motorcycle riders out of the roughly 8.2 million licenses issued in Pennsylvania to operate motor vehicles [7,8]. The sources of power for anti-helmet advocates were clearly rooted in a number of tangible assets: money, organization, people, votes, and offices. The reformers also had a number of intangible sources of political power: information, access to leaders, access to media, and the expertise, skills, and experience necessary to mobilize political allies. All of this led to a political decision on policy form that was in their favor.

## Summary

Was the repeal of the motorcycle helmet law the best public policy decision for the people of Pennsylvania? Public health practitioners and health care professionals tend to be grounded in objective utilitarianism and paternalism, and argue that it is appropriate for government to exert influence over the behavior of its citizens, in selected circumstances, in order to protect the safety and economic viability of our nation as a whole. These decisions, however, must be carefully and thoughtfully researched and the pros and cons seriously considered. Any policy model proposed must be sensitive to the fact that we are a country built upon a culture of individual freedom. However, the choices that we make should not unnecessarily infringe upon the rights of others to enjoy the freedoms that all of us are accustomed to. Motorcyclists involved in preventable crashes that result in death or severe brain injury has an enormous impact on families, friends, co-workers, businesses, economic productivity, and the tax base that we so depend upon for basic human needs. Although easier said than done, political decisions should give equal weight to all of the ethical, political, economic, and cultural perspectives of a particular situation when considering public health policy reforms.

## Abbreviations

NHTSA: National Highway Traffic Safety Administration; MCC: motorcycle crash; CODES: Crash Outcome Data Evaluation System; ABATE: American Bikers Active Towards Education; BATNA: Best Alternative to Negotiated Agreement; SMRO: state motorcycle rights organization.

## Competing interests

The author declares that they have no competing interests.

## Authors' contributions

RAC was solely responsible for the commentary.

## Pre-publication history

The pre-publication history for this paper can be accessed here:

http://www.biomedcentral.com/1471-2458/10/202/prepub
